# A new polymorph of 5-nitro­uracil monohydrate

**DOI:** 10.1107/S1600536808014426

**Published:** 2008-05-17

**Authors:** P. S. Pereira Silva, S. R. Domingos, M. Ramos Silva, J. A. Paixão, A. Matos Beja

**Affiliations:** a CEMDRX, Physics Department, University of Coimbra, P-3004-516 Coimbra, Portugal

## Abstract

In the title compound, C_4_H_3_N_3_O_4_·H_2_O, mol­ecules of 5-nitro­­uracil are hydrogen bonded in pairs across crystallographic centers of symmetry. The resulting dimers are also hydrogen bonded to the water mol­ecules, forming a three-dimensional network. The pyrimidine ring is almost planar (with a maximum deviation of 0.0156 (9) Å for the one of the N atoms) and the nitro group is rotated by 12.4 (1)° out of the uracil plane, while in the other polymorph the value for the same angle is 5°.

## Related literature

For the non-linear optical properties of 5-nitro­uracil, see: Bergman *et al.* (1972 ▶); Puccetti *et al.* (1993 ▶); Youping *et al.* (1992 ▶). For the crystal structure of another polymorph, see: Craven (1967 ▶). For related literature, see: Pettier & Byrn (1982 ▶); Rao *et al.* (1995 ▶).
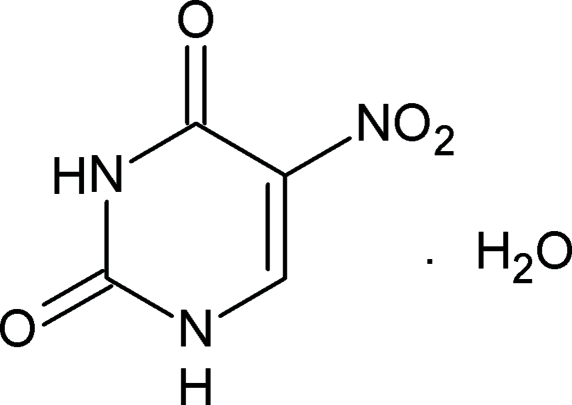


## Experimental

### 

#### Crystal data

C_4_H_3_N_3_O_4_·H_2_O
*M* *_r_* = 175.11
Monoclinic, 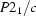

*a* = 6.2799 (1) Å
*b* = 7.8481 (2) Å
*c* = 13.8068 (3) Å
β = 93.842 (1)°
*V* = 678.94 (3) Å^3^
*Z* = 4
Mo *K*α radiation
μ = 0.16 mm^−1^
*T* = 293 (2) K
0.44 × 0.22 × 0.20 mm

#### Data collection

Bruker APEXII CCD area-detector diffractometer
Absorption correction: multi-scan (*SADABS*; Sheldrick, 2003 ▶) *T*_min_ = 0.890, *T*_max_ = 0.969
15135 measured reflections
2232 independent reflections
1918 reflections with *I* > 2σ(*I*)
*R*_int_ = 0.018

#### Refinement

*R*[*F* ^2^ > 2σ(*F* ^2^)] = 0.042
*wR*(*F* ^2^) = 0.131
*S* = 1.00
2232 reflections
115 parameters
H atoms treated by a mixture of independent and constrained refinement
Δρ_max_ = 0.35 e Å^−3^
Δρ_min_ = −0.37 e Å^−3^

### 

Data collection: *APEX2* (Bruker, 2005 ▶); cell refinement: *SAINT* (Bruker, 2005 ▶); data reduction: *SAINT*; program(s) used to solve structure: *SHELXS97* (Sheldrick, 2008 ▶); program(s) used to refine structure: *SHELXL97* (Sheldrick, 2008 ▶); molecular graphics: *PLATON* (Spek, 2003 ▶); software used to prepare material for publication: *SHELXL97*.

## Supplementary Material

Crystal structure: contains datablocks global, I. DOI: 10.1107/S1600536808014426/bt2713sup1.cif

Structure factors: contains datablocks I. DOI: 10.1107/S1600536808014426/bt2713Isup2.hkl

Additional supplementary materials:  crystallographic information; 3D view; checkCIF report

## Figures and Tables

**Table 1:** Hydrogen-bond geometry (Å, °)

*D*—H⋯*A*	*D*—H	H⋯*A*	*D*⋯*A*	*D*—H⋯*A*
N3—H3⋯O2^i^	0.86	1.99	2.8503 (13)	173
N1—H1⋯O9^ii^	0.86	1.88	2.6736 (12)	153
O9—H9*A*⋯O4^iii^	0.87 (2)	1.92 (2)	2.7640 (13)	165 (2)
O9—H9*A*⋯O7^iii^	0.87 (2)	2.41 (3)	2.9101 (13)	117 (2)
O9—H9*B*⋯O7^iv^	0.84 (3)	2.29 (3)	3.0940 (15)	162 (2)

Symmetry codes: (i) 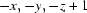; (ii) 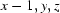; (iii) 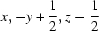; (iv) 
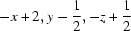
.

**Fig. 1.**
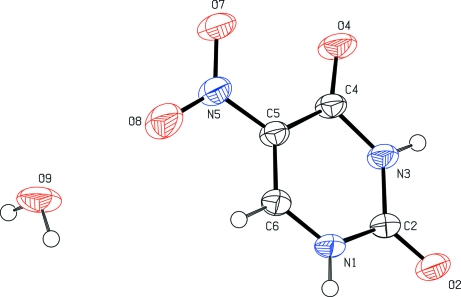
ORTEPII (Spek,2003) plot of the title compound. Displacement ellipsoids are drawn at the 50% probability level.

**Fig. 2.**
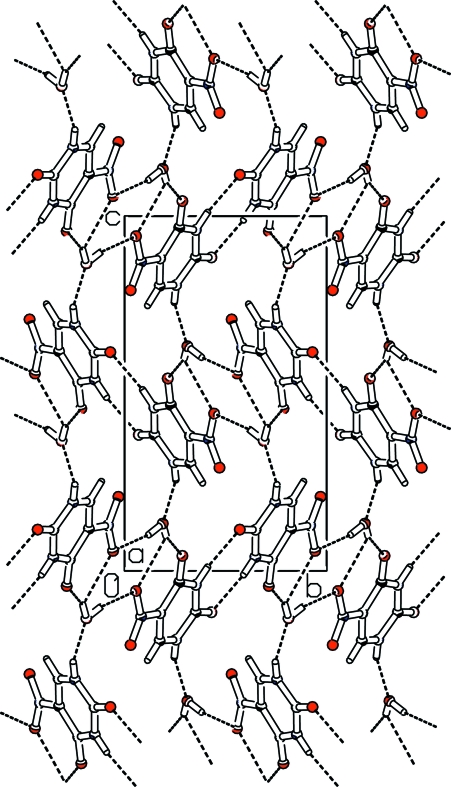
Packing diagram, viewed down the a axis, with the hydrogen bonds depicted as dashed lines.

## supplementary crystallographic information

### Comment

5-Nitrouracil (5NU) is an interesting molecule due to its nonlinear optical properties (Bergman *et al.*, 1972; Puccetti *et al.*, 1993; Youping *et al.*, 1992) and is also of relevance to biological and pharmaceutical sciences (Rao *et al.*, 1995; Pettier & Byrn, 1982).

In the framework of our study of new compounds with potential NLO properties, we have crystallized a new polymorph of 5-nitrouracil monohydrate (Fig.1). Molecules of 5NU are hydrogen bonded in pairs across crystallographic centers of symmetry. The resulting dimers are also hydrogen bonded by the water molecules forming a three dimensional network (Table 1; Fig. 2). The difference in intermolecular interactions between the two polymorphs seems to have a small effect on the molecular structure of the 5NU moiety, with all bond lengths and angles being in good agreement with the previously reported structure (Craven, 1967). The pyrimidine ring is almost planar and the main conformational difference is the large twist of the nitro group away from the plane of the ring [12.4 (1)°], whereas it approaches coplanarity in the other polymorph (5.0° for the same angle).

### Experimental

The title compound was prepared by adding 5-nitrouracil (Aldrich, 98%, 1 mmol) to *L*-serine (Aldrich 99%, 1 mmol) in a solution of water (10 ml) and piridine (Aldrich 99%, 30 ml). The solution was slowly warmed and then left to evaporate under ambient conditions. After a few days, small colourless single crystals were deposited.

### Refinement

All hydrogen atoms were located in a difference Fourier synthesis at an intermediate stage of the refinement. Hydrogen atoms bonded to N and C were placed at calculated positions and refined as riding on their parent atoms, using *SHELXL97* (Sheldrick, 2008) defaults [C—H = 0.93 Å, N—H = 0.86 Å and *U*_iso_(H) = 1.2*U*_eq_(C,*N*)]. The coordinates of the hydrogen atoms of the water molecule were refined with *U*_iso_ = 1.5*U*_eq_(O).

### Crystal data

**Table:**

C_4_H_3_N_3_O_4_·H_2_O	*F*_000_ = 360
*M**_r_* = 175.11	*D*_x_ = 1.713 Mg m^−^^3^
Monoclinic, *P*2_1_/*c*	Mo *K*α radiation λ = 0.71073 Å
Hall symbol: -P 2ybc	Cell parameters from 8093 reflections
*a* = 6.2799 (1) Å	θ = 3.0–33.5º
*b* = 7.8481 (2) Å	µ = 0.16 mm^−^^1^
*c* = 13.8068 (3) Å	*T* = 293 (2) K
β = 93.842 (1)º	Block, colourless
*V* = 678.94 (3) Å^3^	0.44 × 0.22 × 0.20 mm
*Z* = 4	

### Data collection

**Table:**

Bruker APEXII CCD area-detector diffractometer	2232 independent reflections
Radiation source: fine-focus sealed tube	1918 reflections with *I* > 2σ(*I*)
Monochromator: graphite	*R*_int_ = 0.018
*T* = 293(2) K	θ_max_ = 33.6º
φ and ω scans	θ_min_ = 3.0º
Absorption correction: multi-scan(SADABS; Sheldrick, 2003)	*h* = −9→9
*T*_min_ = 0.890, *T*_max_ = 0.969	*k* = −10→11
15135 measured reflections	*l* = −20→20

### Refinement

**Table:**

Refinement on *F*^2^	Secondary atom site location: difference Fourier map
Least-squares matrix: full	Hydrogen site location: inferred from neighbouring sites
*R*[*F*^2^ > 2σ(*F*^2^)] = 0.042	H atoms treated by a mixture of independent and constrained refinement
*wR*(*F*^2^) = 0.131	*w* = 1/[σ^2^(*F*_o_^2^) + (0.0782*P*)^2^ + 0.1592*P*] where *P* = (*F*_o_^2^ + 2*F*_c_^2^)/3
*S* = 1.00	(Δ/σ)_max_ = 0.002
2232 reflections	Δρ_max_ = 0.35 e Å^−^^3^
115 parameters	Δρ_min_ = −0.37 e Å^−^^3^
Primary atom site location: structure-invariant direct methods	Extinction correction: none

### Special details

**Table:**

Geometry. All e.s.d.'s (except the e.s.d. in the dihedral angle between two l.s. planes) are estimated using the full covariance matrix. The cell e.s.d.'s are taken into account individually in the estimation of e.s.d.'s in distances, angles and torsion angles; correlations between e.s.d.'s in cell parameters are only used when they are defined by crystal symmetry. An approximate (isotropic) treatment of cell e.s.d.'s is used for estimating e.s.d.'s involving l.s. planes.
Refinement. Refinement of *F*^2^ against ALL reflections. The weighted *R*-factor *wR* and goodness of fit *S* are based on *F*^2^, conventional *R*-factors *R* are based on *F*, with *F* set to zero for negative *F*^2^. The threshold expression of *F*^2^ > σ(*F*^2^) is used only for calculating *R*-factors(gt) *etc*. and is not relevant to the choice of reflections for refinement. *R*-factors based on *F*^2^ are statistically about twice as large as those based on *F*, and *R*- factors based on ALL data will be even larger.

### Fractional atomic coordinates and isotropic or equivalent isotropic displacement parameters (Å^2^)

**Table:**

	*x*	*y*	*z*	*U*_iso_*/*U*_eq_	
O2	−0.10857 (13)	0.07025 (12)	0.38353 (6)	0.0403 (2)	
O4	0.51917 (15)	0.21379 (14)	0.54232 (6)	0.0478 (3)	
O7	0.75926 (14)	0.44337 (13)	0.44352 (7)	0.0459 (2)	
O8	0.67663 (18)	0.46987 (19)	0.29111 (8)	0.0674 (4)	
N1	0.11205 (14)	0.24517 (12)	0.30605 (6)	0.0306 (2)	
H1	0.0210	0.2540	0.2569	0.037*	
N3	0.20903 (15)	0.14746 (12)	0.46071 (6)	0.0330 (2)	
H3	0.1773	0.0894	0.5106	0.040*	
N5	0.63828 (16)	0.41810 (13)	0.37147 (7)	0.0371 (2)	
C2	0.06017 (16)	0.14825 (13)	0.38398 (7)	0.0290 (2)	
C4	0.40561 (16)	0.22918 (14)	0.46790 (7)	0.0309 (2)	
C5	0.44553 (16)	0.32373 (13)	0.38070 (7)	0.0292 (2)	
C6	0.29851 (16)	0.32614 (14)	0.30377 (7)	0.0307 (2)	
H6	0.3287	0.3858	0.2481	0.037*	
O9	0.90537 (17)	0.18878 (17)	0.13340 (7)	0.0555 (3)	
H9A	0.790 (4)	0.206 (3)	0.0966 (18)	0.083*	
H9B	0.975 (4)	0.119 (3)	0.1021 (18)	0.083*	

### Atomic displacement parameters (Å^2^)

**Table:**

	*U*^11^	*U*^22^	*U*^33^	*U*^12^	*U*^13^	*U*^23^
O2	0.0332 (4)	0.0481 (5)	0.0381 (4)	−0.0115 (3)	−0.0090 (3)	0.0058 (3)
O4	0.0397 (5)	0.0639 (6)	0.0370 (4)	−0.0138 (4)	−0.0176 (3)	0.0143 (4)
O7	0.0378 (5)	0.0521 (5)	0.0457 (5)	−0.0136 (4)	−0.0137 (4)	0.0035 (4)
O8	0.0525 (6)	0.1010 (10)	0.0474 (6)	−0.0336 (6)	−0.0066 (4)	0.0276 (6)
N1	0.0274 (4)	0.0381 (4)	0.0251 (4)	−0.0006 (3)	−0.0067 (3)	0.0008 (3)
N3	0.0301 (4)	0.0406 (5)	0.0271 (4)	−0.0066 (3)	−0.0066 (3)	0.0064 (3)
N5	0.0308 (4)	0.0408 (5)	0.0387 (5)	−0.0065 (4)	−0.0055 (3)	0.0064 (4)
C2	0.0272 (4)	0.0313 (4)	0.0278 (4)	−0.0005 (3)	−0.0046 (3)	−0.0010 (3)
C4	0.0278 (5)	0.0353 (5)	0.0286 (4)	−0.0025 (4)	−0.0067 (3)	0.0020 (3)
C5	0.0259 (4)	0.0324 (5)	0.0285 (4)	−0.0023 (3)	−0.0040 (3)	0.0017 (3)
C6	0.0292 (5)	0.0358 (5)	0.0264 (4)	0.0003 (4)	−0.0030 (3)	0.0022 (3)
O9	0.0458 (5)	0.0762 (7)	0.0413 (5)	0.0151 (5)	−0.0218 (4)	−0.0152 (5)

### Geometric parameters (Å, °)

**Table:**

O2—C2	1.2234 (12)	N3—C4	1.3888 (13)
O4—C4	1.2169 (11)	N3—H3	0.8600
O7—N5	1.2268 (13)	N5—C5	1.4320 (13)
O8—N5	1.2206 (14)	C4—C5	1.4502 (14)
N1—C6	1.3345 (13)	C5—C6	1.3601 (13)
N1—C2	1.3745 (13)	C6—H6	0.9300
N1—H1	0.8600	O9—H9A	0.87 (2)
N3—C2	1.3651 (12)	O9—H9B	0.84 (3)
			
C6—N1—C2	122.44 (8)	N3—C2—N1	115.09 (9)
C6—N1—H1	118.8	O4—C4—N3	118.84 (9)
C2—N1—H1	118.8	O4—C4—C5	128.88 (10)
C2—N3—C4	127.78 (9)	N3—C4—C5	112.29 (8)
C2—N3—H3	116.1	C6—C5—N5	117.19 (9)
C4—N3—H3	116.1	C6—C5—C4	120.62 (9)
O8—N5—O7	122.28 (10)	N5—C5—C4	122.19 (9)
O8—N5—C5	118.20 (10)	N1—C6—C5	121.72 (9)
O7—N5—C5	119.52 (9)	N1—C6—H6	119.1
O2—C2—N3	123.33 (9)	C5—C6—H6	119.1
O2—C2—N1	121.57 (9)	H9A—O9—H9B	104 (2)
			
C4—N3—C2—O2	179.00 (11)	O7—N5—C5—C4	−11.85 (17)
C4—N3—C2—N1	−1.51 (16)	O4—C4—C5—C6	179.88 (12)
C6—N1—C2—O2	−177.51 (10)	N3—C4—C5—C6	0.19 (15)
C6—N1—C2—N3	2.99 (15)	O4—C4—C5—N5	−0.29 (19)
C2—N3—C4—O4	−179.72 (11)	N3—C4—C5—N5	−179.97 (10)
C2—N3—C4—C5	0.00 (16)	C2—N1—C6—C5	−2.99 (16)
O8—N5—C5—C6	−12.45 (17)	N5—C5—C6—N1	−178.58 (10)
O7—N5—C5—C6	168.00 (11)	C4—C5—C6—N1	1.27 (17)
O8—N5—C5—C4	167.71 (13)		

### Hydrogen-bond geometry (Å, °)

**Table:**

*D*—H···*A*	*D*—H	H···*A*	*D*···*A*	*D*—H···*A*
N3—H3···O2^i^	0.86	1.99	2.8503 (13)	173
N1—H1···O9^ii^	0.86	1.88	2.6736 (12)	153
O9—H9A···O4^iii^	0.87 (2)	1.92 (2)	2.7640 (13)	165 (2)
O9—H9A···O7^iii^	0.87 (2)	2.41 (3)	2.9101 (13)	117 (2)
O9—H9B···O7^iv^	0.84 (3)	2.29 (3)	3.0940 (15)	162 (2)

Symmetry codes: (i) −*x*, −*y*, −*z*+1; (ii) *x*−1, *y*, *z*; (iii) *x*, −*y*+1/2, *z*−1/2; (iv) −*x*+2, *y*−1/2, −*z*+1/2.
